# Supplementary material for: Summative evaluation of the rural surgical obstetrical networks initiative: Findings from a five year retrospective qualitative study
Source: PLoS One. 2026 Mar 17;21(3):e0334388. doi: 10.1371/journal.pone.0334388 (PMC12994810; doi:10.1371/journal.pone.0334388)
Supplement: S1 File — (PDF) [file pone.0334388.s001.pdf]

Evaluation of Rural Surgical and Obstetrical Networks (RSON) in BC
Interview guide – Year 4 Summative

Interview with RCCbc/pillar leads key stakeholders

Purpose: understand and document points of interaction between RSON and the health systems, how those interactions went and in retrospect how it could have been done better; documentation and advice; perception of network function.

Background and Context:

1. What is your role in RSON?
2. How did your position come about (invited in, seconded, etc.)?
3. What background prepared you for this role?
4. What kind of support did you have in your role?
5. What experience did you have working in networks?
6. Tell us about the relationships you developed with members of the HA:
    a. Did you start off productively as you wish you could have?
    b. Were there any points of contention early on? [If yes]
        i. How did you resolve them?
        ii. Were there any notables wins/successes? Describe them.
7. What were the main challenges implementing RSON?
8. How did you manage them?

Pillar leads:

9. How did you feel about the rollout of your pillar?
    a. Was there enough guidance?
    b. Was there a clear plan?
    c. Did the plan need to be adjusted? Why?
    d. What were some of the most sign challenges in rolling out the pillar?
    e. What were some of the successes?
    f. What worked really well?

Ongoing management and direction

10. Did you have a clear workplan and set of goals and objectives for the as you were wrapping up RSON? [If yes]

11. What were the main of your goals/objectives?
12. Did anything interfere with meeting your goals and objective?
13. What strategies did you develop to mitigate?
14. What kind of support do you have? What kind of support do you need?
15. What do you see as the most important achievements that RSON has made?

16. Have you experienced any internal conflict in your team? With the cental RSON team? [If yes]
    a. How was it resolved?
    b. How would you have liked it to be resolved?
17. What was your role in strategic planning?
18. Would you have liked more or less of a role in strategic planning?
19. How much autonomy do you feel you had with budget decisions for the budget allocated to your pillar?
20. Did you think decision making processes were transparent? Did you feel included?

21. Do you think there was a cohesive understanding of the network vision and how to accomplish it at your site?
22. Do you think your reciprocal referral/rural site shares this vision?
23. Can you describe the relationship between your site and the reciprocal referral/rural site?
    a. What aspects of this relationship went/are going well?
    b. What aspects of this relationship could be improved?
24. What was the biggest "win" of RSON at your site?
25. Was there any place where RSON fell short?
26. What will the legacy of RSON be at your site?
27. What part of the initiative is most important to retain after the formal part of the initiative is over?
28. Is there anything about RSON that is important to understand that we haven't talked about yet?
