# Supplementary material for: Summative evaluation of the rural surgical obstetrical networks initiative: Findings from a five year retrospective qualitative study
Source: PLoS One. 2026 Mar 17;21(3):e0334388. doi: 10.1371/journal.pone.0334388 (PMC12994810; doi:10.1371/journal.pone.0334388)
Supplement: S2 File — (PDF) [file pone.0334388.s002.pdf]

**S2 File. ThoughtExchange question 1 responses**

| Respondent ID | What were the gains and benefits of RSON at your site ? | Star score - overall | Rank - overall |
|---|---|---|---|
| Respondent 1 | Increasing and maintaining surgical and maternity services so that patients are able to stay in their home towns for their procedures | 4.3 | 1 |
| Respondent 2 | Flexible Coaching and Mentoring (Peer and in larger centres) Encourages our rural practicioners to build relationships and comfort in conducting procedures they don't do often. | 4.3 | 2 |
| Respondent 3 | Maternity sustainability - all over the province programs are closing | 4.3 | 3 |
| Respondent 4 | Increased local focus on maternity and OR. We made it through Covid-19 without losing our surgical or maternity services. | 4.3 | 4 |
| Respondent 5 | Coaching from regional and provincial providers keep skills fresh in low volume sites | 4.3 | 5 |
| Respondent 6 | New services in our OR, supported by education and quality initiatives | 4.3 | 6 |
| Respondent 7 | Building confidence - Many coaching opportunities | 4.2 | 7 |
| Respondent 8 | Coaching opportunities from local colleagues and from specialists at urban sites | 4.2 | 8 |
| Respondent 9 | The research findings are so encouraging - boosts our cred[ibility]! We knew it was there. Hopefully the [health authorities] and [ministry of health] honour that. | 4.2 | 9 |
| Respondent 10 | A focus on maternity. It's an underfunded department that doesn’t get enough attention for the huge impact it has on our community | 4.2 | 10 |
| Respondent 11 | Building confidence in physician and midwifery skills. Promoting strong leadership | 4.2 | 11 |
| Respondent 12 | Helping with coaching. Helps build our skills and team to ensure best patient outcomes | 4.2 | 12 |
| Respondent 13 | It provided a structure and forum to keep our OR and Mat program alive! Maternity care close to home for so many women. | 4.2 | 13 |
| Respondent 14 | Strengthened our surgical and maternity services. Helped us create a cohesive team dynamic. Allowed time for quality care initiatives. Without RSON, our maternity services would likely have collapsed. We have learnt the value of regular team meetings and peer to peer coaching. | 4.2 | 14 |

| | | | |
|---|---|---|---|
| Respondent 15 | Team collaboration and communication. We cannot work together in any other way! | 4.2 | 15 |
| Respondent 16 | Educational funding!!! So nurses and doctors can get paid to be better at their jobs. Much more realistic than expecting voluntary, altruistic education which burns out | 4.2 | 16 |
| Respondent 17 | Funding to keep nurses and physicians working together for common goals to maintain the program and make it more sustainable. Sustainability is easier to recruit into | 4.2 | 17 |
| Respondent 18 | Improving connections with other rural sites. | 4.2 | 18 |
| Respondent 19 | Increased surgical scope with associated coaching and support to improve provider confidence and retention | 4.2 | 19 |
| Respondent 20 | Learning from other communities | 4.2 | 20 |
| Respondent 21 | Linking with other programs maternity and surgery are interdependent. RSON helped encourage that | 4.2 | 21 |
| Respondent 22 | Team building increased substantially by including ALL our team members and remuneration for their time. Everyone in the OR team is important, RSON helped bring the whole group together locally and regionally | 4.2 | 22 |
| Respondent 23 | Dedicated coaching funds To value team | 4.2 | 23 |
| Respondent 24 | RSON has provided a number of educational opportunities and improved work environments through its funding. Funding provides opportunity to seek educational opportunities that might not be feasible to attend. It's been amazing having RSON funded lines. | 4.2 | 24 |
| Respondent 25 | Connections outside of our hospital walls | 4.1 | 25 |
| Respondent 26 | Integral to keeping maternity services at our site. It gave us the space and flexibility to design a system that works for us | 4.1 | 26 |
| Respondent 27 | Team communication. Building a team improves knowledge sharing | 4.1 | 27 |
| Respondent 28 | Team work | 4.1 | 28 |
| Respondent 29 | Teamwork | 4.1 | 29 |
| Respondent 30 | Increase scope of procedures increased numbers and networks is more resilient | 4.1 | 30 |
| Respondent 31 | Building & strengthening relationships | 4.1 | 31 |

| | | | |
|---|---|---|---|
| Respondent 32 | We have expanded our program and done some amazing quality work. The quality work has helped us improve patient care in all areas | 4.1 | 32 |
| Respondent 33 | 24/7 OR. Keep maternity going | 4.1 | 33 |
| Respondent 34 | Huge impact on expanding our local OR scope and volume. Prior to RSON our OR ran 1.5 days/week, RSON gave us the tools to build up to 4.5d/wk. Familiarity in the OR and repetition has allowed our team to work effectively, efficiently and be better prepared for emergencies. | 4.1 | 34 |
| Respondent 35 | Increased skill development of nurses. Confidence in practice | 4.1 | 35 |
| Respondent 36 | Dedication to quality | 4.1 | 36 |
| Respondent 37 | Establishing mentorship relationships amongst staff Peer to peer learning and collaboration to learn as a team | 4.1 | 37 |
| Respondent 38 | Relationship building between Health Authority Quality Patient Safety structures and processes with RSON. | 4.1 | 38 |
| Respondent 39 | Administrative capacity to support regional and provincial networking and local RSON activities. Not supported elsewhere in [health authority] so this localized support was highly valuable. | 4 | 39 |
| Respondent 40 | Education. Improve the quality of service provided | 4 | 40 |
| Respondent 41 | Education opportunity, retention, job satisfaction | 4 | 41 |
| Respondent 42 | Increased communication between OR & OB teams. | 4 | 42 |
| Respondent 43 | Needs assessments done with OB RN team. So we know what to work on and how to do it | 4 | 43 |
| Respondent 44 | Stable staffing in the OR. Improved clinical skills | 4 | 44 |
| Respondent 45 | Team work. Collaboration between providers | 4 | 45 |
| Respondent 46 | Teamwork. Better understanding from each other | 4 | 46 |
| Respondent 47 | Collaboration of our interdisciplinary teams towards mutual goals Created a sense of community, camaraderie and made us so much more resilient when Covid hit our teams were so strong, because we supported each other | 4 | 47 |
| Respondent 48 | Integration with other maternity support programs collective supports for success and reduce silos | 4 | 48 |
| Respondent 49 | LDR tracking tool. So we can objectively assess our outcomes | 4 | 49 |

| | | | |
|---|---|---|---|
| Respondent 50 | Links with other resources and hearing what other sites are working on. Shared ideas and challenges | 4 | 50 |
| Respondent 51 | Support for new staff. Promoting maternity care locally. | 4 | 51 |
| Respondent 52 | Team Work New staff and communication | 4 | 52 |
| Respondent 53 | Team development Improved care | 4 | 53 |
| Respondent 54 | Time dedicated to Quality improvements | 4 | 54 |
| Respondent 55 | Working Group is a forum for Administration, Staff, and physicians to talk about Surgical Sustainability. Important to discuss as a team rather than in silos. | 4 | 55 |
| Respondent 56 | bridging the silos of surgery and maternity services They go hand in hand but prior to RSON, there was some us vs them attitudes. Now people understand how they are both integral. | 4 | 56 |
| Respondent 57 | team culture all team members contribution to care | 4 | 57 |
| Respondent 58 | Education, training, mentoring and coaching Improved confidence and competence leading to better patient outcomes | 4 | 58 |
| Respondent 59 | It allowed for increased communication with various staff and physicians on various topics. | 4 | 59 |
| Respondent 60 | Opportunity to run Education skills day for teams We're able to encourage more participants with the availability of funding for education | 4 | 60 |
| Respondent 61 | Independence from the purse strings of the health authority allowed us the agility to pivot and fund quality initiatives locally. Through trial and error we were able to run through multiple sequential PDSA cycels of CQI. Thus able to find successes quicker. | 4 | 61 |
| Respondent 62 | Stabilized Maternity care Maternity care close to home is vital to the health of women and communities. | 4 | 62 |
| Respondent 63 | Access to supports and research as well as creating a larger network for the site and practitioners Rural has significant challenges and needs | 3.9 | 63 |
| Respondent 64 | Engaging multidisciplinary team with flexibility | 3.9 | 64 |
| Respondent 65 | New positions like cqi nurse | 3.9 | 65 |
| Respondent 66 | Opportunity to focus on quality improvement Leads to better care | 3.9 | 66 |

| | | | |
|---|---|---|---|
| Respondent 67 | Collegiality To learn and respect each other's professions and better understand the work involved in everyone's role | 3.9 | 67 |
| Respondent 68 | Great to have accurate, easily accessible data to help us optimize our program Need accurate data in order to optimize program, know what we are actually doing, what are we doing well, what can we improve upon | 3.9 | 68 |
| Respondent 69 | covid anaesthesia difficult during COVID. RSON helped fund this | 3.9 | 69 |
| Respondent 70 | data to validate safety and quality helps keep everyone and the regional sites respecting the role of the small surgical sites | 3.9 | 70 |
| Respondent 71 | improved team relationships and communication | 3.9 | 71 |
| Respondent 72 | increased volume of OB, surgical opportunities, allowed for educational experience to brush up on surgical skills sustains competency for rural providers, support for new/younger staff/providers | 3.9 | 72 |
| Respondent 73 | Increased OR capacity and thus increasing utilization with expansion of service, coaching key part of this Supports competency and enhances patient care | 3.9 | 73 |
| Respondent 74 | Improving policies and procedures Being up to date and efficient | 3.8 | 74 |
| Respondent 75 | Tools - surveys, data, rtvs | 3.8 | 75 |
| Respondent 76 | scope and volume sustainablity over time | 3.8 | 76 |
| Respondent 77 | Increase of OR services Amazing how we have gone from a quiet OR site to a much busier department simply through some funding and an opportunity to try | 3.8 | 77 |
| Respondent 78 | Our site changed immensely. We gained staff, positions, equipment, increase in FTE's, and able to implement amazing projects to better our site. To continue our sites growth and learning. | 3.8 | 78 |
| Respondent 79 | creating communication networks between referring centres and rural sites supports rural care providers and enhances knowledge of what is done in rural sites for FRCP referral sites | 3.8 | 79 |
| Respondent 80 | A platform to engage and motivate passionate stakeholders morale and sustainability | 3.7 | 80 |
| Respondent 81 | Telehealth equipment | 3.7 | 81 |
| Respondent 82 | Increased staffing to stabilize and sustain services | 3.7 | 82 |

| | | | |
|---|---|---|---|
| Respondent 83 | a CQI position that helped us to organize our maternity department Addressed deficiencies that no one else had previously had "responsibility" for, kept education as a priority and organized the department | 3.7 | 83 |
| Respondent 84 | service stability preventing service disruption or diversion | 3.6 | 84 |
| Respondent 85 | Beginnings of working with health authority We are the tailOf the dog in our health authority! We'd love to be more front and centre | 3.6 | 85 |
| Respondent 86 | Local coordinators They keep us on track. Being local is important | 3.6 | 86 |
| Respondent 87 | We were given administrative, CQI, and financial support to focus on what's important to our local team. We felt valued and heard, the local team got to decide what to work on without having to worry about all the administrative work in the background. | 3.6 | 87 |
| Respondent 88 | Increase in staffing and positions We needed regular positions to keep the OR open, cannot rely on casual | 3.4 | 88 |
| Respondent 89 | Increased OR days, OR staffing, types of surgeries, medical equipment Strengthened the OR in capacity to provide quality care in a rural site with long commutes, provision of mat support, even emergency care | 3.3 | 89 |
| Respondent 90 | Increased funding for educational opportunities, team coaching, workshops, and ability to obtain feedback on areas lacking confidence This has raised our knowledge base, confidence, competence in providing obstetrical/perioperative care in rural site | 3.3 | 90 |
| Respondent 91 | Provision of financial and human resources, networking capacity to support quality improvement initiatives Allows the facility to level up their care by focusing on what's important to them, networking to see what works at other sites, get new ideas | 3.3 | 91 |
| Respondent 92 | education,confidence and team building positive support and skills building | 3.3 | 92 |
| Respondent 93 | upgraded our aging equipment, We need to have working equipment to perform our job | 3.2 | 93 |
