# Supplementary material for: Summative evaluation of the rural surgical obstetrical networks initiative: Findings from a five year retrospective qualitative study
Source: PLoS One. 2026 Mar 17;21(3):e0334388. doi: 10.1371/journal.pone.0334388 (PMC12994810; doi:10.1371/journal.pone.0334388)
Supplement: S3 File — (PDF) [file pone.0334388.s003.pdf]

S3 File. ThoughtExchange question 2 responses

Respondent ID	In an ideal world, which are the most crucial interventions to sustain surgical & obstetrical	Star score - overall	Rank - overall
Respondent 1	Continue to expand surgical services at rural sites to tackle weight lists and to provide rural sites with the	4.4	1
Respondent 2	Staffing for maternity nursing and OR nursing - It’s	4.3	2
Respondent 3	Coaching and mentoring	4.3	3
Respondent 4	Maintain and continue to improve scope and volume key to maintenance of healthy and safe OR	4.3	4
Respondent 5	Flexible funding for the local surgical and obstetric team's time and CQI which is independent of the health authority bureaucracy. Each site is unique and	4.3	5
Respondent 6	Retention of maternity RNs. We can not deliver out patients here if they are not here - feeling safe /	4.3	6
Respondent 7	Scope and Volume support for rural OR programs helps sustain rural maternity and OR services	4.3	7
Respondent 8	[The RSON Project Manager]	4.3	8
Respondent 9	Appropriate interdisciplinary staffing levels	4.2	9
Respondent 10	Local coordinator	4.2	10
Respondent 11	Coaching/training/simulations to build competency	4.2	11
Respondent 12	Continued support for appropriately trained staff and staffing levels. Ongoing education	4.2	12
Respondent 13	Education is needed to sustain services. Without trained nurses programs will fail. Also need increased	4.2	13
Respondent 14	Safe staffing levels so everyone is supported	4.2	14
Respondent 15	Staffing. We do not have maternity nurses.	4.2	15
Respondent 16	Volume of care/experiences, learning from other's experience, compensation for this financially. It's the only way small communities can maintain high quality	4.2	16
Respondent 17	Adequate Staffing . Without trained staff cannot	4.2	17
Respondent 18	Increased staffing that we received to ensure patient and staff safety - it has made such a difference, the	4.2	18
Respondent 19	Recruitment and retention of perinatal trained nurses that are trained to work in a rural setting, and supporting those nurses to maintain their skill. We	4.2	19

Respondent 20	Funding to sustain the amazing cohesive teams that already exist and to provide care close to home	4.2	20
Respondent 21	Increase surgical capacity to ensure ORs stay open and continue with maternity education to increase confidences, retention and recruitment. Open ORs =	4.2	21
Respondent 22	Coaching.<br>Sustain skills and develops new ones current with the	4.1	22
Respondent 23	Diversifying surgical services. Creates a robust program, retains and engages staff and gives the team	4.1	23
Respondent 24	Staff and Education To help support and feel	4.1	24
Respondent 25	Coaching interdisciplinary coaching, specialists	4.1	25
Respondent 26	site specific budgets for education/skills enhancement important for rural to have designated supports so as	4.1	26
Respondent 27	Appropriate nursing levels. Safety, retention, provider	4.1	27
Respondent 28	Coaching opportunities for the full team. Support on going education and supports future development, the team again have felt so supported with coaching and	4.1	28
Respondent 29	Stable baseline staffing. Creates capacity for things	4.1	29
Respondent 30	[The RSON Project Manager]!!	4.1	30
Respondent 31	Interdisciplinary coaching team building, improved skills, networking with regional sites	4.1	31
Respondent 32	Tailored education, APP, on-going coaching and mentorship, safe staffing levels Competency,	4.1	32
Respondent 33	Supporting surgeons at rural sites, keeping jobs- job security, supporting jobs to make it happen, rural hospital having procedures that can admit to keep the	4.1	33
Respondent 34	Continued education opportunities<br>Staff confidence and cohesion	4	34
Respondent 35	Guaranteed work and Guaranteed Pay for sustainability of Anesthesia services Scope, Volume, and APP's (as a	4	35
Respondent 36	Recurrent onsite training/SIMS for uncommon	4	36
Respondent 37	Education and practical experience	4	37
Respondent 38	interprofessional education and learning	4	38
Respondent 39	The local coordinator positions are key	4	39

Respondent 40	Interdisciplinary coaching<br>Support and consistency throughout the site and for	4	40
Respondent 41	Embrace a culture of learning and embedded quality improvement at the local level	3.9	41
Respondent 42	Ongoing education and collaborative interagency work	3.9	42
Respondent 43	Team based care - Fosters great interdisciplinary	3.9	43
Respondent 44	Integration of surgery and maternity keeps or going and integrates teams for more sustainability of the site	3.9	44
Respondent 45	Administrative support	3.9	45
Respondent 46	Funding for mentorship/coaching/Sims	3.9	46
Respondent 47	Nurse coaching	3.9	47
Respondent 48	Funding for SIMS allows uncommon critical events in rural areas to be reviewed in a multidisciplinary	3.9	48
Respondent 49	Opportunities for team communication and collaboration	3.8	49
Respondent 50	Access to locums	3.8	50
Respondent 51	Funding for more OR time! Hard to sell the need for local priorities to health authority	3.8	51
Respondent 52	Supported funding for Education. This is vital for recruitment and retention of skilled and knowledgeable	3.8	52
Respondent 53	Coordinators someone to put all the pieces of generalism together on the ground to help the teams	3.8	53
Respondent 54	Team based care	3.8	54
Respondent 55	Coordinator<br>Keep the scope and volume work alive	3.8	55
Respondent 56	Funding for expanding/maintaining education.<br>Increased sustainability for both maternity and surgical	3.8	56
Respondent 57	Funding for coaching creates more confident and safer	3.8	57
Respondent 58	[The RSON Project Manager]!!!!	3.8	58
Respondent 59	Team support!! Having safe staffing levels and providing ongoing education for all staff. You would have better communication, someone to provide	3.8	59
Respondent 60	Supporting education and ongoing competency for RN's<br>RN's in smaller sites do not have access to ongoing	3.8	60

Respondent 61	The positions: educator, LCC, CQI lead. Builds	3.7	61
Respondent 62	Quality - no other quality programs our there for small	3.7	62
Respondent 63	APP	3.7	63
Respondent 64	Protected time for Quality Improvement work<br>The amazing work that has been done by the local QI teams has been instrumental for sustaining all the	3.7	64
Respondent 65	Structured format to organize initiatives and manage funding: project manager, LCC, CQI nurse<br>Efficiency and to keep the burden off front line staff	3.7	65
Respondent 66	Our local working group! Keeps us connected and working towards the same goals.	3.7	66
Respondent 67	CQI lead on site	3.6	67
Respondent 68	Local CQI support	3.6	68
Respondent 69	Virtual technology minimizes the challenges of	3.6	69
Respondent 70	Continuous quality improvement work	3.6	70
Respondent 71	To have site-based coordinator. To ensure timely coordination and who knows the site and works with	3.6	71
Respondent 72	Coaching and CQI work coaching supports the confidence and quality of care for patients at rural sites. CQI develops and captures the improvements within	3.6	72
Respondent 73	Paid educational/coaching support<br>Support for education beyond NRP/FHS in maternity, coaching to share knowledge, experience higher	3.6	73
Respondent 74	QI and data acquisition - Allows team to support each other with knowledge acquisition and target specific	3.6	74
Respondent 75	Skills sessions, confidence with education	3.6	75
Respondent 76	We need to have the staff to provide these services.<br>We need to have a nice work environment if people are	3.5	76
Respondent 77	Expanded OR services -- new surgeons, new procedures which made our OR team more robust.<br>Expanded our list of procedures so that our team was learning new things, and keeping engaged. Also, [illegible]	3.5	77
Respondent 78	Having a central rural coordinator continue ability to share ideas and have a rural cohesive approach to	3.4	78

Respondent 79	Regional Coordinator - To support data collection, equitable access to care, connection to rural provincial	3.4	79
Respondent 80	Staff the OR adequately - Reduces gaps in call so there is consistent surgical back-up	3.3	80
Respondent 81	Funding, education and resources. With these in place I believe we can continue to support the current services we are providing. We need sustainability of the Rural Surgical Obstetrics Network (RSON) in order to [illegible]	3.3	81
