# Supplementary material for: Summative evaluation of the rural surgical obstetrical networks initiative: Findings from a five year retrospective qualitative study
Source: PLoS One. 2026 Mar 17;21(3):e0334388. doi: 10.1371/journal.pone.0334388 (PMC12994810; doi:10.1371/journal.pone.0334388)
Supplement: S4 File — (DOCX) [file pone.0334388.s004.docx]

**S4 File. Data visualizations.**

Data Visualization A. Number of comments by theme across merged ThoughtExchanges.


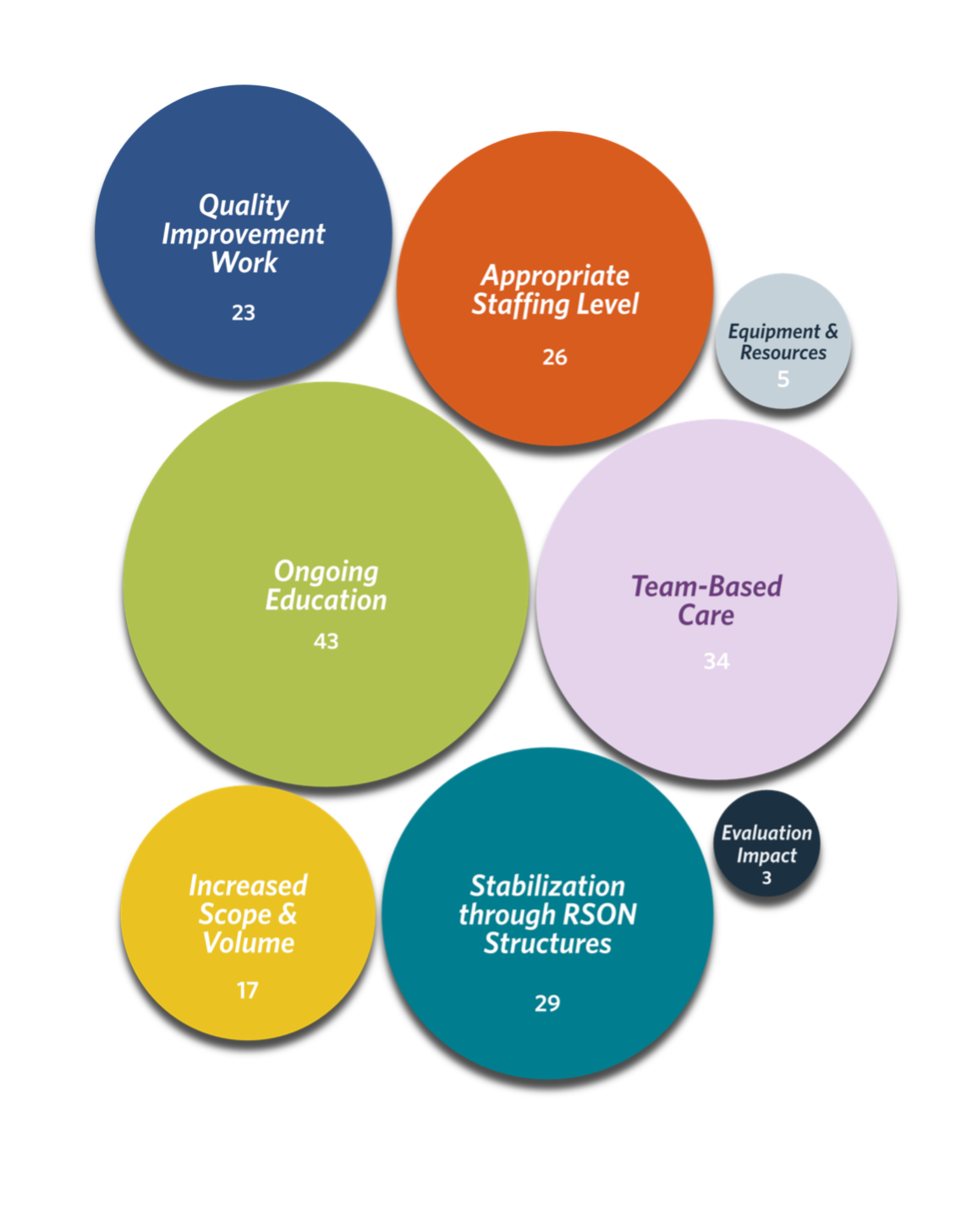


Data Visualization B. Average Star rating of comments organized by theme.

Following comment sharing in the ThoughtExchange platform, participants were able to anonymously rate other comments out of five stars. Comments were organized by theme, and the average star rating by theme is represented below.

**
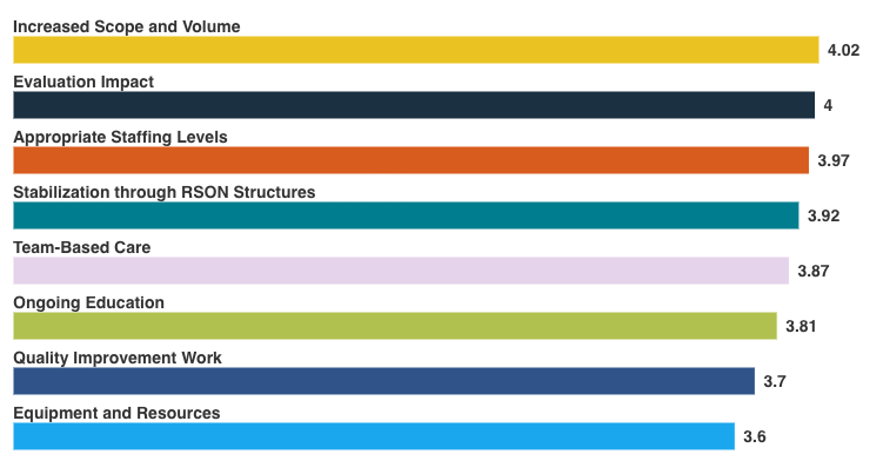
**
